# Victorian Institute of Sport Assessment questionnaire specifically tailored for greater trochanteric pain syndrome for the Dutch population

**DOI:** 10.1093/jhps/hnae026

**Published:** 2024-08-28

**Authors:** Damien Van Quickenborne, Catherine Van Der Straeten, Arne Burssens, Emmanuel Audenaert

**Affiliations:** ANCA Clinic Ghent, Xavier De Cocklaan 68/1, Deurle 9831, Belgium; Orthopaedic Department, Ghent University Hospital, Corneel Heymanslaan 10, Ghent 9000, Belgium; Orthopaedic Department, Ghent University Hospital, Corneel Heymanslaan 10, Ghent 9000, Belgium; Orthopaedic Department, Ghent University Hospital, Corneel Heymanslaan 10, Ghent 9000, Belgium; Orthopaedic Department, Ghent University Hospital, Corneel Heymanslaan 10, Ghent 9000, Belgium

## Abstract

Greater trochanteric pain syndrome (GTPS) is a highly prevalent condition characterized by lateral hip and thigh pain. The Victorian Institute of Sport Assessment (VISA) questionnaire specifically tailored for GTPS (VISA-G) questionnaire was developed for the purpose of assessing and quantifying the severity of symptoms related to gluteal tendinopathy or GTPS. It is commonly used in research and clinical settings to evaluate the impact of GTPS on patient function and quality of life. The VISA-G questionnaire was developed for English-speaking populations. Before this questionnaire can be used in non-English-speaking populations, it has to be translated and validated for a particular population. The current study aimed to translate and validate the VISA-G questionnaire for a Dutch-speaking context (VISA-G-Dutch). In this study, we conducted a comprehensive process involving forward and back translation, along with a thorough comparison with other established hip-related questionnaires. The COSMIN checklist was used to ensure uniformity in the validation study. A sample of 100 participants, 50 symptomatic and 50 asymptomatic, completed the VISA-G-Dutch, Harris Hip Score, Hip Disability and Osteoarthritis Outcome Score, Oxford Hip Score, and Nonarthritic Hip Score questionnaires. Internal consistency and test–retest reliability were measured. Construct validity was assessed through positive correlations between the VISA-G-Dutch and gold standard questionnaires. Strong internal consistency and test–retest reliability correlations were found in both the asymptomatic and symptomatic groups. The test–retest reliability also demonstrated strong positive correlations for the symptomatic group. The standard error of measurement was ∼2.3 for the symptomatic group. These results prove that the VISA-G-Dutch is a reliable and valid tool for assessing GTPS and gluteal tendinopathy in Dutch-speaking individuals, providing clinicians with a valuable assessment tool.

## Introduction

Greater trochanteric pain syndrome (GTPS) manifests as pain in the lateral hip and thigh and is also identified as trochanteric bursitis, along with tendinopathy or tearing of the gluteus medius (Gmed) or gluteus minimus (Gmin) muscles [1]. In 2014, Fearon *et al*. [2] developed the Victorian Institute of Sport Assessment (VISA) questionnaire specifically tailored for GTPS, referred to as VISA-G. The primary objective of the VISA-G is to track patient outcomes and assess the efficacy of treatment strategies for individuals dealing with GTPS. The questionnaire model employs item response theory, with graded responses indicating increasing difficulty [3].

The VISA-G questionnaire was translated into multiple languages (German, Portuguese, Turkish, French, and Danish) and validated, indicating the global recognition and significance of GTPS as a distinct pathology. This global reach allows researchers and clinicians from different regions to utilize the VISA-G questionnaire in their respective languages to assess and manage patients with GTPS effectively [4–6].

Patient-reported outcome measures are used to measure function and pain and have gained prominence in contemporary clinical practice, particularly in evaluating the effectiveness of orthopedic surgeries treatments, especially for patients treated by arthroplasty surgery [7, 8]. Currently, there is a lack of a dedicated tool for assessing GTPS, as the commonly used questionnaires in orthopedic hip surgery are geared toward hip arthroplasty and are not suitable for GTPS evaluation [9, 10].

Subjective scoring systems designed as questionnaires can be adapted for use in different countries by translating and validating them for the target language population. The primary objective of our study was to translate the VISA-G for Dutch-speaking populations through both forward and back translation processes [8]. Our goal was to investigate whether there were any significant differences in the outcomes of the VISA-G questionnaire when compared to other established reference questionnaires in assessing patients with GTPS.

We hypothesized that there would be no significant difference in the outcomes of the VISA-G questionnaire between the original version and the Dutch-speaking version for patients with GTPS.

## Material and methods

### Translation procedure

The English version of the VISA-G was translated into Dutch using the translation procedure according to the guidelines of cross-cultural adaptation from Guillemin *et al*. [11]; permission to do so was obtained from the developer of the original VISA-G [2]. This process involved forward translation by two Dutch-speaking translators, for synthesis, and back translation by two native English speakers, expert panel review, pilot testing with GTPS patients, and cultural adaptation if necessary. This rigorous method ensured linguistic accuracy for the Dutch-speaking context.

The forward translation and data acquisition were performed by two bilingual native Dutch (mother tongue) speakers. Both had no medical training. The translation was also reviewed and corrected by an online translating tool (Google Translate™; translate.google.com). A 2021 study conducted by the UCLA Medical Center found that Google Translate preserved the overall meaning in 82.5% of the translations [12]. The result was the first version of the VISA-G-Dutch.

**Figure 1.**
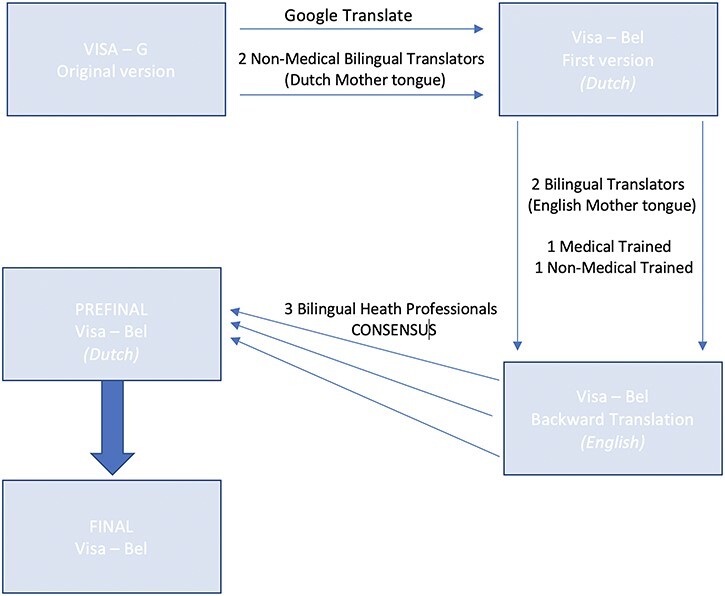
Translation procedure diagram.

As seen in Fig. 1, the backward translation was subsequently done by two other bilingual translators with English as their mother tongue, who were blinded to the original version of the VISA-G and who independently translated the VISA-G-Dutch questionnaire back into English. One of the translators had medical training.

Following this, three health professionals met to consolidate all versions of the questionnaire and develop what would be considered the prefinal version (VISA-G-Dutch) of the questionnaire for field testing. This committee reviewed all translations and reached a consensus on any discrepancies.

The last phase was to test this VISA-G-Dutch questionnaire on a sample of participants (*n* = 20) with GTPS. The purpose was to investigate how the participants interpreted each item and to ensure that no remaining language issues were present in the questionnaire. Comments on clarity of wording and problems encountered during the self-administration process were collected.

After these steps, we considered this as our final version of the VISA-G Dutch.

To ensure the accuracy of translations, it is crucial to consult several sources for cross-referencing. As such, we compared the translations of the original VISA-G questionnaire with those generated by Google Translate to verify the precision of our final version (VISA-G Dutch), which were found to be highly accurate [12].

### VISA-G and VISA-G Dutch

The VISA-G and VISA-G Dutch (final translated version, as described earlier) consist of eight questions (Supplementary Appendix 1). The first question relates to hip pain in general and is scored on a scale from 0 to 10 (most pain = 0; no pain = 10). The remaining seven questions relate to everyday activities and participation. These questions measure the participants’ ability to lie on the sore hip, walk on stairs, walk on ramps or slopes, move from sitting to walking, work around the house or garden, participate in regular exercise, and weight-bearing-related pain and function. Question 8 is divided into three subsections, of which only one section is required to be answered. The final score is calculated out of 100. The higher the score out of 100 the less disability perceived by the individual [2, 9].

### Harris Hip Score, Hip Disability and Osteoarthritis Outcome Score, and Oxford Hip Score

The outcome measures used in this study, together with the translated version of the VISA-G-Dutch questionnaire, were the Harris Hip Score (HHS), Hip Disability and Osteoarthritis Outcome Score (HOOS), and the Oxford Hip Score (OHS). These are considered the gold standards in our practice to evaluate the painful hips.

We are aware of the fact that these questionnaires are used for patients with total hip replacement (THR) or with osteoarthritis (OA) of the hip, but they provide a good evaluation of quality of life (QOL) for individuals with a painful hip.

The HHS is a validated health-related quality of life (HRQL) instrument. It was developed for the assessment of the results of hip surgery and is intended to evaluate various hip disabilities and methods of treatment in an adult population. The original version was published in 1969. The maximum score is 100 [13, 14].

The HOOS was designed to assess patients’ perceptions regarding their hip replacement. It is easy to complete and provides an option to examine changes in pain, other symptoms, activities of daily living (ADLs), sports and recreation, as well as hip-related QOL. The HOOS consists of five subscales: pain, other symptoms, function in ADLs, function in sports and recreation (sport/rec), and hip-related QOL. The maximum score is 100 on all five subscales [13].

The OHS was designed to assess the outcome after THR by measuring patients’ perceptions regarding their hip surgery. The original version from 1996 was updated in 2007, introducing a new scoring system. The OHS assesses pain (six items) and function (six items) of the hip in relation to daily activities, such as walking, dressing, and sleeping. The maximum score is 48 [13].

### Nonarthritic Hip Score

The outcome measures mentioned earlier are all tools for assessing the patient outcomes after arthroplasty or in cases of OA of the hip. The Nonarthritic Hip Score (NAHS) [15] is a clinical assessment tool used to evaluate hip function in individuals without arthritis. It focuses on assessing pain, function, and patient satisfaction following hip-related injuries or conditions that do not involve OA [14]. The NAHS questionnaire consists of a series of questions addressing various aspects of hip function, including pain levels, mobility, ADLs, and overall QOL. By quantifying these factors, the NAHS provides healthcare professionals with valuable information to guide treatment decisions and monitor the progress of nonarthritic hip conditions. The maximum score of the NAHS is 100. In brief, the NAHS [15] can be used as a measurement tool that was not developed for patients with hip arthroplasty, unlike the VISA-G.

### Population

Based on an expected reliability of 0.90, assuming a power of 0.80 (Type II error) and a significance level of 0.05 (Type I error), by using the intraclass correlation coefficient (ICC) analysis, we calculated that a total sample size of 100 patients would be sufficient and 50 would be required for test–retest reliability calculation (50 symptomatic and 50 asymptomatic patients). This sample size is similar to that used in other orthopedic questionnaire validation articles [16–18].

We selected a consecutive group of 50 native Dutch-speaking GTPS patients in our clinic, they were symptomatic patients with obvious clinical signs of GTPS who were asked to participate in filling out our questionnaire (VISA-G-Dutch). After screening for exclusion criteria (rheumatoid arthritis, coxarthrosis, bone-related disorders, previous hip surgery, specific comorbidities that could affect physical activity, and/or a 3-month use of corticosteroids), the VISA-G-Dutch questionnaire was handed to these 50 participants. They were also asked to fill out a scoring form for the HHS, HOOS, OHS, and NAHS (Table 1).

**Table 1.** Population characteristics (age, gender, and included total = *n*)

	Asymptomatic	Symptomatic
Included total (*n*)	50	50
Age (years), mean (±SD)	53 (±11.2)	56 (±9.8)
Gender (M/F), *n* (%)	32 (64)/18 (36)	17 (34)/33 (66)
Age (years), mean (SD)	63 (6.12)	57 (2.17)
Side (L/R) (%)	68/32	54/46

A control group of 50 native Dutch-speaking asymptomatic participants was recruited through social media in Belgium (Ghent) using the same exclusion criteria as the symptomatic group. By placing a simple advertisement on Instagram and Facebook, we quickly found the necessary candidates. All participants were properly informed about the study goals and the setup and signed an informed consent form. The study was approved by the local ethics committee (MS/FB/AVDS/2022/22).

### Statistical analysis

#### Validity

Construct validity of the VISA-G-Dutch was evaluated by calculating the Pearson correlation coefficient (*r*) to measure the strength of the correlations between the scores of different scales and subscales. A correlation of *r* > 0.5 was considered strong [11]. We acknowledge the fact that the HHS, HOOS, OHS, and NAHS currently are the gold standards to assess hip problems. This study also evaluates how the VISA-G-Dutch scores relate to this gold standard. We therefore calculated the Pearson correlation coefficient (*r*) to examine the correlation between the gold standards and the VISA-G-Dutch questionnaire. A correlation coefficient of <0.2 was considered as weak, between 0.2 and 0.4 as acceptable, between 0.4 and 0.6 as good, and >0.6 as strong [7].

Content validity of a questionnaire is a nonstatistical term and relies more on expert opinion to determine whether the scale covers a representative sample of the domain to be measured [19]. The scores of the VISA-G-Dutch were compared with nonparametric statistical tests (Mann–Whitney U-test and Kruskal–Wallis test) and also assessed against other validated measures such as the HHS, HOOS, OHS, and NAHS (paired *t*-tests). The occurrence of floor effects (a large number of subjects with the poorest score of 0) and ceiling effects [17, 20] (a large number of subjects reaching the best score of 100) was also examined to evaluate the ability of the VISA-G-Dutch to distinguish further improvements and its responsiveness to smaller effect changes. These effects occur when a questionnaire repetitively scores the maximum or minimum value and represent a measuring limitation because they indicate a questionnaire may not be set properly to what it is intended to measure [12].

#### Test–retest reliability

Test–retest reliability is a measure of the consistency of test scores over time. It is used to determine whether a test yields consistent results when it is administered to the same group of people at different times. The test–retest reliability coefficient or the ICC is calculated by administering the same test twice to a group of individuals at two different time points and then correlating the two sets of scores. For the second assessment, we asked for responses to be submitted within a timeframe of 7 days, the latest answer was after 11 days [18]. Paired *t*-tests were performed to determine the systematic difference between the first and second tests. We have performed a retest only with the symptomatic group (*n* = 50).

Generally, a correlation coefficient (an ICC) of ≥0.70 is considered to indicate good test–retest reliability [21].

#### Internal consistency

Internal consistency is defined as the degree of interrelatedness among the items of a scale and is measured with the Cronbach’s alpha (CA) coefficient [22]. This coefficient ranges from 0 to 1, with higher values indicating higher internal consistency. It has been recognized that a value between 0.7 and 0.9 reflects good internal consistency [16, 23]. To measure internal consistency, we first measured the global alpha coefficient for the VISA-G-Dutch questionnaire.

#### Standard error of measurement

The standard error of measurement (SEM) refers to the amount of error that is inherent in a measurement. It is a measure of the degree of reliability or consistency of a test or measurement. The SEM is calculated as the standard deviation of the scores of a test or measurement divided by the square root of the number of items or observations in the test or measurement.

In general, a smaller SEM indicates greater reliability and precision of a test or measurement, while a larger SEM indicates greater variability and less precision. Therefore, minimizing SEM is a key goal in test development and measurement. We calculated the SEM following the COSMIN guidelines [3].

Because patients were asked to simultaneously complete four different scores containing many similar questions, there was a risk of overwhelming the patients, which could lead to missing responses.

## Results

### Total score

The VISA-G-Dutch scores for the asymptomatic group (*n* = 50) ranged from 84 to 100 out of 100 (mean = 96; SD 3.95/median 92), the retest assessment was similar (mean = 97; SD 3.95/median 93). The VISA-G-Dutch scores for the symptomatic GTPS patients (*n* = 50) ranged from 43 to 78 out of 100 (mean = 62; SD 6.65). Scoring results for both groups, according to the HHS, HOOS, OHS, and NAHS are summarized in Table 2.

**Table 2.** VISA-G-Dutch, HHS, HOOS (separate scores), OHS, and NAHS for the asymptomatic and symptomatic groups

Scores	Asymptomatic (*n* = 50 , mean (SD/range)	Symptomamtic (*n* = 50 , mean (SD/range)
VISA-G-Dutch (0–100)	96 (3.95/84–100)	62 (6.65/43–78)
HHS (0–100)	97	82
HOOS pain	98	66
HOOS symptoms	94	72
HOOS function	98	66
HOOS sport/rec	96	76
HOOS QOL	88	78
OHS (0–48)	44	32
NAHS (0–100)	96	58

There was some noncompliance among patients in the symptomatic group, with two individuals not responding and not completing the retest questionnaires within the specified 1-week interval.

### Construct validity

Overall, the VISA-G-Dutch correlated strongly with the reference questionnaires (HHS, HOOS, OHS, and NAHS). Through this correlation analysis, our goal was to benchmark the VISA-G-Dutch’s ability to measure hip problems effectively. It is important to note that our assessment was conducted separately for the asymptomatic and symptomatic groups to ensure a comprehensive understanding of construct validity in different patient populations. The best correlation we found was with the HOOS (total) score in both the asymptomatic and symptomatic groups (*r* = 0.87; *P* < .001/*r* = 0.90; *P* < .001). The other scores (HHS, OHS, and NAHS) also had excellent significant (*P* < .001) correlations (Tables 3 and 4).

**Table 3.** Pearson correlation factor (*r*) between VISA-G-Dutch and HHS, HOOS (total), OHS, and NAHS for the asymptomatic group

VISA-G-Dutch (asymptomatic)	HHS	HOOS (total)	OHS	NAHS
Pearson correlation factor (*r*)	0.82 (*P* < .001)	0.87 (*P* < .001)	0.78 (*P* < .001)	0.86 (*P* < .001)

**Table 4.** Correlation (Pearson correlation factor/*r*) between VISA-G-Dutch and HHS, HOOS (total), OHS, and NAHS for the symptomatic group.

VISA-G-Dutch (symptomatic)	HHS	HOOS (total)	OHS	NAHS
Pearson correlation factor (*r*)	0.88 (*P* < .001)	0.90 (*P* < .001)	0.84 (*P* < .001)	0.89 (*P* < .001)

Because of the highest correlation with the HOOS (total), we additionally tested the construct validity of the five HOOS subscales: pain (*r* = 0.89; *P* < .001), other symptoms (*r* = 0.83; *P* < .001), function in ADLs (*r* = 0.87; *P* < .001), function in sports and recreation (*r* = 0.79; *P* < .001), and hip-related QOL (*r* = 0.88; *P* < .001). Each subscale showed that there was a significant correlation with the VISA-G-Dutch.

Content validity was proven by a significant difference between symptomatic and asymptomatic patients (*P* < 0.001) consistent with the HHS, HOOS, OHS, and NAHS, which also differed significantly between the asymptomatic and symptomatic groups [7]. Floor and ceiling effects did not occur in the symptomatic group. However, in the asymptomatic group, a clear ceiling effect was observed with all scores >90.

### Test–retest reliability and internal consistency

Of the 50 patients in the symptomatic group, only 48 returned both questionnaires (test + retest). The mean interval was <1 week (mean of 6.2 days, with a range of 2–17 days) (Table 5). The test–retest for the symptomatic group had great reliability, with a positive correlation (ICC) between test and retest (symptomatic *n* = 48, *r* = 0.87; *P* < .001). The SEM for the symptomatic group was 2.3, which is considered good for a questionnaire with a maximum score of 100. Internal consistency, as indicated by CA ranging from .86 to .97 for the symptomatic group and the separate questions, was good to excellent. For the total score, the CA was .96, which is considered excellent.

**Table 5.** Test–retest reliability ICC and internal consistency CA for the total score of the Visa-G-Dutch and its domains.

VISA-G-Dutch symptomatic	Test 1 (*n* = 50), (mean/SD)	Test 2 (*n* =48), (mean/SD)	ICC	CA
Question 1 (10–0)	3 (1.0)	3 (1.2)	0.90	.96
Question 2 (10–0)	3 (1.5)	2 (1.0)	0.84	.87
Question 3 (10–0)	4 (1.5)	4 (1.6)	0.90	.96
Question 4 (10–0)	3.5 (1.2)	3 (1.0)	0.86	.94
Question 5 (10–0)	2 (1.0)	2 (1.2)	0.93	.97
Question 6 (10–0)	3 (1.4)	3.2 (1.6)	0.86	.91
Question 7 (10–0)	4 (1.6)	3.5 (1.2)	0.84	.89
Question 8 (A, B, and C), (0–90)	38 (11)	41 (13)	0.81	.86
Visa-G-Dutch total (0–100)	62/6.65	64/7.75	0.87 (*P* < .001)	.96 (*P* < .001)

## Discussion

The study aimed to translate and validate the original VISA-G questionnaire for Dutch-speaking populations, resulting in the VISA-G-Dutch. The translation process encountered no difficulties, as all items in the original questionnaire were clearly understood and interpreted by both independent translators and patients. Since the questionnaire’s items were deemed universally applicable to all patients, there was no need for cultural adaptation during the translation procedure. In summary, the study effectively translated and validated the VISA-G questionnaire for the Dutch-speaking population, ensuring its clarity and relevance in a different linguistic context [11].

We would like to acknowledge specific limitations of our study. First, our data collection for test–retest scores was limited to questionnaires from patients in the symptomatic group, which may compromise the accuracy of our test–retest results due to the smaller sample size (*n* = 50). Second, the administration of five questionnaires simultaneously, each containing similar items, may have overwhelmed the participants, resulting in varied responses to identical questions. Third, there was minimal noncompliance among patients, with two individuals not responding and not completing the retest questionnaires within the specified 1-week interval. However, these few limitations are outweighed by the strengths of the study. The internal consistency of the VISA-G-Dutch questionnaire was assessed using CA, which yielded a very good consistency score of .96 for the symptomatic group. The test–retest reliability also demonstrated strong positive correlations for this group (symptomatic: *r* = 0.87; *P* < .001). These findings indicate that the translated questionnaire, VISA-G-Dutch, consistently measures the intended constructs over time and demonstrates good reliability. The study also demonstrated good content validity of the VISA-G-Dutch questionnaire. The SEM found in the symptomatic group, which estimates the imprecision or variability in test scores due to random error, was ∼2.3, which is an excellent result for a questionnaire with a maximum score of 100.

We found excellent construct validity and a strong correlation between the VISA-G-Dutch and the reference questionnaires for hip pathology (HHS, HOOS, OHS, and NAHS). The questionnaires were categorized into the two known groups: asymptomatic and symptomatic patients. The asymptomatic group showed high scores across all domains. In contrast, the symptomatic group had lower scores, reflecting more significant impairments in hip function, pain, and QOL. Furthermore, we found a positive correlation between the VISA-G-Dutch questionnaire and the gold standard questionnaires in both asymptomatic and symptomatic groups. These strong correlations (Pearson correlation coefficients ranging from 0.78 to 0.90) demonstrate the questionnaire’s ability to measure similar constructs as the established measures, further supporting its validity.

The results of this study validated the translation of the VISA-G questionnaire into the VISA-G-Dutch and its effectiveness in assessing symptom severity and functional outcomes.

## Conclusion

The Dutch version of the VISA-G, the VISA-G-Dutch, is a reliable and valid instrument for assessing the severity of GTPS and gluteal tendinopathy among Dutch-speaking individuals and is a valuable tool for Dutch-speaking clinicians.

The psychometric properties of the Dutch version were assessed through a series of analyses, including internal consistency, test–retest reliability, and construct validity.

The practical value of the new VISA-G-Dutch will need to be demonstrated when clinicians incorporate it into their daily practice.

In conclusion, this new questionnaire, the VISA-G-Dutch, can be used as a reliable tool for Dutch-speaking GTPS patients.

## Supplementary Material

hnae026_Supp

## Data Availability

The data underlying this article will be shared on reasonable request to the corresponding author.
