# Supplementary material for: Victorian Institute of Sport Assessment questionnaire specifically tailored for greater trochanteric pain syndrome for the Dutch population
Source: J Hip Preserv Surg. 2024 Aug 28;11(4):257–62. doi: 10.1093/jhps/hnae026 (PMC11744470; doi:10.1093/jhps/hnae026)
Supplement: hnae026_Supp [file hnae026_supp.zip › suppl_data/APPENDIX1-2.pdf]

ANCA CLINIC

VISA-G

Gelieve één vakje aan te kruisen voor elke vraag. Kies het vakje dat het beste met jouw probleem overeenkomt – het moet niet perfect zijn. Alle vragen hebben betrekking op je pijn aan de heup.

Vraag 1 : mijn heuppijn is :

Geen pijn → 1 2 3 4 5 6 7 8 9 10 → meest pijn

Vraag 2 : kan ik op mijn pijnlijke heup liggen ?

- Voor langer dan 1 uur
- Voor 30 minuten tot 1 uur, dan moet ik bewegen
- Voor 15 tot 30 minuten, dan moet ik bewegen
- Voor 5 tot 15 minuten, dan moet ik bewegen
- Ik kan helemaal niet op mijn pijnlijke heup liggen

Vraag 3 : Een trap op en af lopen

- Ik kan een trap gebruiken zonder pijn te voelen
- Ik kan een trap gebruiken met beperkte heuppijn
- Ik kan een trap gebruiken met behulp van een leuning wegens de heuppijn
- Ik kan een trap gebruiken stap voor stap met gebruik van de leuning door de heuppijn
- Ik kan een trap niet gebruiken wegens de heuppijn

Vraag 4 : Een helling op en af lopen

- Ik kan normaal een helling oplopen zonder heuppijn
- Ik kan een helling oplopen met lichte heuppijn
- Ik heb lichte moeilijkheden een helling op te lopen door de heuppijn
- Ik heb grote moeilijkheden om een helling op te lopen door de heuppijn
- Ik kan een helling niet oplopen vanwege de heuppijn

Vraag 5 : Na 30 minuten neerzitten is opstaan en wandelen :

- Geen probleem
- Moeilijk voor enkele stappen
- Ik moet een ogenblik stilstaan vooraleer te wandelen
- Ik moet tenminste 20 seconden stilstaan vooraleer ik kan wandelen
- Ik moet meer dan 20 seconden stilstaan vooraleer ik kan wandelen

Vraag 6 : Huishoudelijke taken en werken in de tuin

- Ik kan in mijn huis of mijn tuin werken voor meer dan 1 uur
- Omwille van de heuppijn kan ik in huis of in de tuin tussen 30 en 60 min werken
- Omwille van de heuppijn doe ik zeer weinig werk in het huis of de tuin
- Omwille van de heuppijn doe ik alleen werk in huis en niet in de tuin
- Omwille van de heuppijn kan ik geen werk doen in huis of de tuin

Vraag 7 : Doet U momenteel oefeningen, fysieke activiteiten of sport ?

- Ja, ik kan oefeningen doen zoals ik gewoon ben
- Een ietsje minder dan ik gewoon was
- Aanzienlijk minder dan ik gewoon was
- Neen, ik kan geen oefeningen doen, ik wil dit niet, ik heb er de tijd niet voor

Vraag 8 : drie onderdelen, gelieve enkel sectie A, B of C te beantwoorden

Beïnvloedt Uw actuele heuppijn Uw mogelijkheid om bepaalde activiteiten uit te voeren ?

Onderdeel A : Mijn heuppijn is zo erg dat ik niet langer kan wandelen, shoppen, of lopen

Als dit zo is, hoeveel van deze activiteiten doet U elke dag ?

- Ik doe geen bijkomende activiteiten, ik beweeg enkel in mijn huis
- Ik doe minder dan 10 min activiteiten
- Ik doe 10 – 19 minuten
- Ik doe 20 – 29 minuten
- Ik doe meer dan 30 minuten

Onderdeel B : Ik voel heuppijn bij oefeningen, maar het weerhoudt mij er niet van om te wandelen, te shoppen, te lopen of andere vormen van oefening uit te voeren.

Als dit zo is, hoeveel van deze activiteiten doet U elke dag ?

- Ik doe geen bijkomende activiteiten, ik beweeg enkel ik mijn huis
- Ik doe minder dan 10 min
- Ik doe 10 -19 min
- Ik doe 20 - 29 min
- Ik doe meer dan 30 min

Onderdeel C : Als U geen pijn voelt bij het wandelen, het shoppen, het lopen, of andere fysieke activiteiten.

Als dit zo is hoeveel van deze activiteiten doet U elke dag ?

- Ik doe geen bijkomende activiteiten, ik beweeg enkel ik mijn huis
- Ik doe minder dan 10 min
- Ik doe 10 -19 min
- Ik doe 20 - 29 min
- Ik doe meer dan 30 min

VISA-G second draft

Please mark one box in each question. Choose the box that best suits you – it may not be perfect. All the questions relate to your HIP pain.

Question 1: My usual hip pain is...

10	9	8	7	6	5	4	3	2	1	0
☐	☐	☐	☐	☐	☐	☐	☐	☐	☐	☐
**0**	**1**	**2**	**3**	**4**	**5**	**6**	**7**	**8**	**9**	**10**
**no pain**										**worst pain**

Question 2: I can lie on my sore hip

10 ☐ For longer than 1 hour

7 ☐ For 30 minutes to 1 hour, then I have to move

5 ☐ For 15 to 30 minutes, then I have to move

2 ☐ For 5 to 15 minutes, then I have to move

0 ☐ I am unable to lie on my sore side at all

Question 3: Walking up or down one flight of stairs

10 ☐ I can use stairs normally with no hip pain

7 ☐ I can use stairs normally with some hip pain

5 ☐ I can use stairs normally holding onto a banister because of hip pain

2 ☐ I use stairs one step at a time and holding onto a banister because of hip pain

0 ☐ I cannot use stairs at all because of hip pain

Question 4: Walking up or down a ramp or slope

10 ☐ I can walk normally up and down a slope or ramp with no hip pain

7 ☐ I can walk normally up and down a slope or ramp with slight hip pain

5 ☐ I have some difficulty walking up and down a slope or ramp because of hip pain

2 ☐ I have significant difficulty negotiating slopes or ramps because of hip pain

0 ☐ I cannot walk up or down a slope or ramp because of hip pain

Question 5: After sitting for 30 minutes, moving to standing and then walking is...

10 ☐ Not a problem

7 ☐ Difficult for a few steps

5 ☐ I have to stand still for a moment or two before I walk

2 ☐ I have to stand still for less than 20 seconds before I walk

0 ☐ I have to stand still for more than 20 seconds before I walk

Question 6: Work about the house or garden (or similar activity)

10 ☐ I can work in my house and/or garden for an hour or more

7 ☐ Because of hip pain, I can work in my house and/or garden in 30 to 60 min bursts

5 ☐ Because of hip pain, I do very limited work in my house and garden

2 ☐ Because of hip pain, I do limited work in my house but I do not garden

0 ☐ Because of hip pain, I do not do any work in my house or garden

Question 7: Are you currently taking part in regular exercise, physical activity or sport?

10 ☐ Yes – I can exercise as I used to.

7 ☐ Somewhat less than I used to.

4 ☐ Significantly less than I used to.

0 ☐ No – I am unable to exercise, I don't want to or I don't have time.

Question 8 has *Three* sections. Please answer section A, B or C ONLY.

Does your current hip pain affect your ability to undertake weight bearing activities? (e.g. walking, shopping, running, squats, lunges).

Section A: My hip pain is so severe that it will stop me from walking, shopping, running or other weight bearing exercise.

If this is so, how much of this activity do you do each day?

0 ☐ I do not undertake any extra activity on my legs - I only move about the house.

2 ☐ I do less than 10 minutes.

5 ☐ I do 10 – 19 minutes.

7 ☐ I do 20 – 29 minutes.

10 ☐ I do more than 30 minutes.

Section B: My hip pain is present with exercise, but it does not stop me from walking, shopping, running or other weight bearing type exercise.

If this is so, how much of this activity do you do each day?

0 ☐ I do not undertake any extra activity on my legs - I only move about the house.

5 ☐ I do less than 10 minutes.

10 ☐ I do 10 – 19 minutes.

15 ☐ I do 20 – 29 minutes.

20 ☐ I do more than 30 minutes.

Section C: If you have no pain while you undertake walking, shopping, running or other weight bearing type exercise.

If this is so, how much of this activity do you do each day?

6 ☐ I do not undertake any extra activity on my legs - I only move about the house.

12 ☐ I do less than 10 minutes.

18 ☐ I do 10 – 19 minutes.

24 ☐ I do 20 – 29 minutes.

30 ☐ I do more than 30 minutes

TOTAL SCORE = /100

Scoring

Q1: 10 - x

Q2-Q6: Top/first option = 10, 2nd = 7, 3rd = 5, 4th =2, 5th option = 0

Q7: Top/first option = 10, 2nd = 7, 3rd = 4, 4th = 0

Q8:

Section A 1st option = 0, 2nd = 2, 3rd = 5, 4th = 7, last/5th option = 10

Section B 1st option = 0, 2nd = 5, 3rd = 10, 4th = 15, last/5th option = 20

Section C 1st option = 6, 2nd = 12, 3rd = 18, 4th = 24, last/5th option = 30
